# Supplementary material for: The limits of near field immersion microwave microscopy evaluated by imaging bilayer graphene moiré patterns
Source: Nat Commun. 2021 May 20;12:2980. doi: 10.1038/s41467-021-23253-2 (PMC8170674; doi:10.1038/s41467-021-23253-2)
Supplement: Supplementary file 1 — Supplementary Information [file 41467_2021_23253_MOESM1_ESM.pdf]

Supplementary Information:

The limits of Near Field Immersion Microwave Microscopy evaluated by imaging bilayer graphene moiré patterns

Douglas A. A. Ohlberg,[1] Diego Tami,[1,2] Andreij C. Gadelha,[3] Eliel G. S. Neto,[4] Fabiano C. Santana,[3] Daniel Miranda,[3] Wellington Avelino,[2] Kenji Watanabe,[5] Takashi Taniguchi,[5] Leonardo C. Campos,[3] Jhonattan C. Ramirez,[2,6] Cássio Gonçalves do Rego,[2,6] Ado Jorio,[2,3,7] and Gilberto Medeiros-Ribeiro* [2,8]

[1] *Microscopy Center, Universidade Federal de Minas Gerais, Belo Horizonte, MG 31270-901, Brazil*

[2] *Electrical Engineering Graduate Program, Universidade Federal de Minas Gerais, Belo Horizonte, MG 31270-901, Brasil.*

[3] *Physics Department, Universidade Federal de Minas Gerais, Belo Horizonte, MG 31270-901, Brazil.*

[4] *Instituto de Física, Universidade Federal da Bahia, Campus Universitário de Ondina, Salvador - BA, 40170-115 Brazil.*

[5] *National Institute for Materials Science (NIMS), 1-2-1 Sengen, Tsukuba-city, Ibaraki 305-0047, Japan.*

[6] *Department of Electronic Engineering, School of Engineering, Universidade Federal de Minas Gerais, Belo Horizonte, MG 31270-901, Brazil*

[7] *Technology Innovation Graduate Program, Universidade Federal de Minas Gerais, Belo Horizonte, MG 31270-901, Brazil.*

[8] *Computer Science Department, Universidade Federal de Minas Gerais, Belo Horizonte, MG 31270-901, Brazil*

* gilberto@dcc.ufmg.br

SUPPLEMENTARY NOTE 1: EXPERIMENTAL SETUP

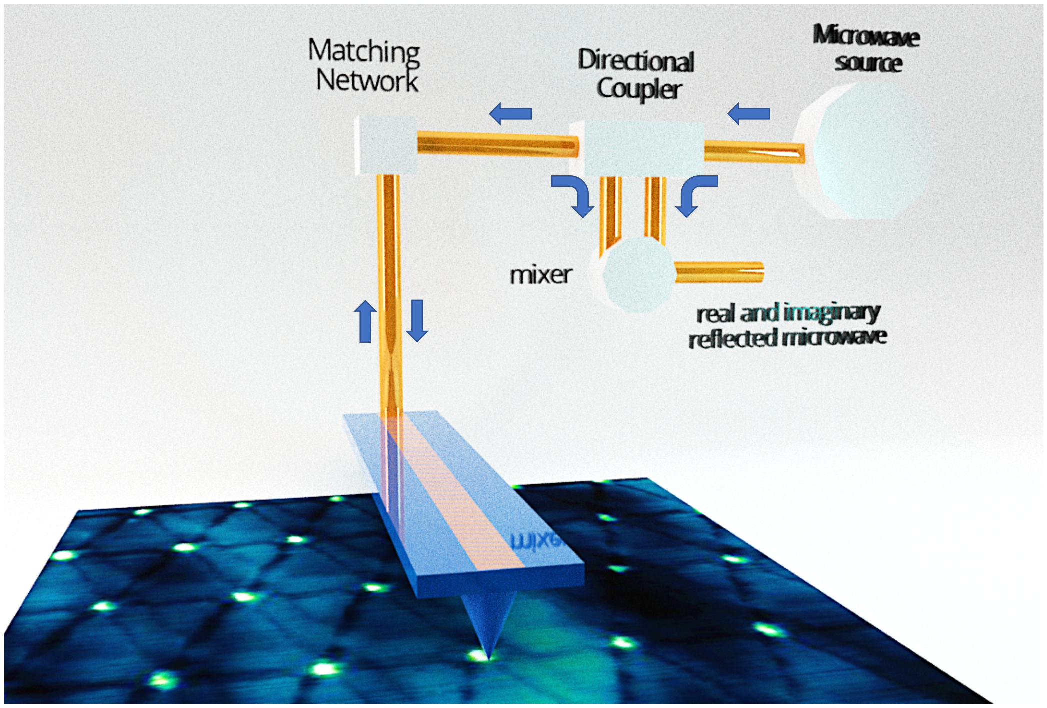

Supplementary Fig. 1. **Experimental set up showing the microwave circuitry for sample probing.**

SUPPLEMENTARY NOTE 2: RAMAN AND SURVEY STRATEGY

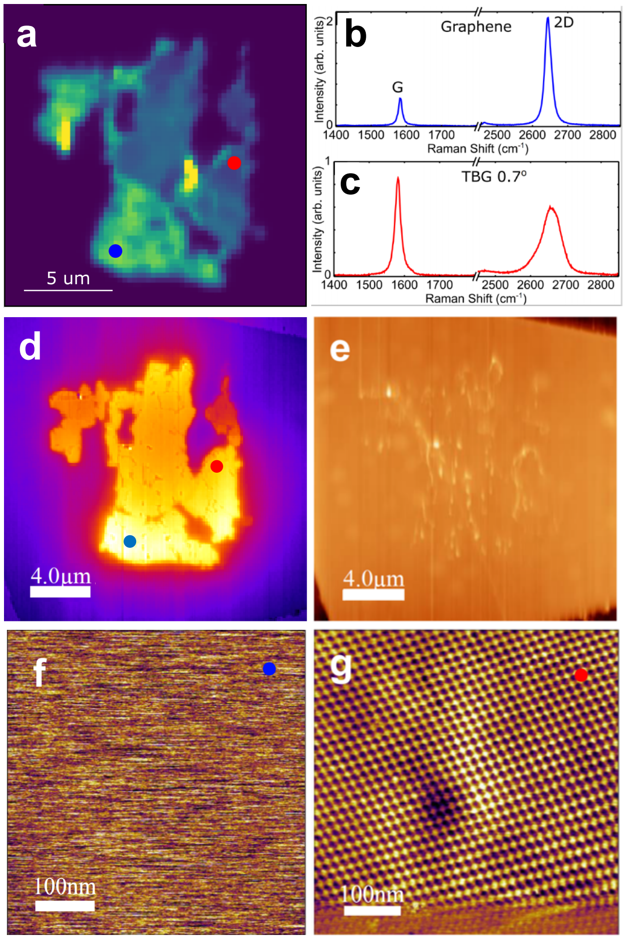

Supplementary Fig. 2. **Survey protocol.** **a**. MicroRaman image of composite graphene flake created by tear and stack process, composed of monolayer and twisted bilayer regions. The color scale is keyed to 2D peak intensity with bright yellow and green corresponding to monolayer regions and dark blue to twisted bilayer regions. **b**., **c**. Raman spectra taken over monolayer and bilayer sites indicated, respectively, in **a**. by blue and red dots. **d**. Capacitance image (imaginary part of the reflected microwave) of same composite flake (**a**.). **e**. Atomic Force Microscopy Topography image simultaneously acquired with capacitance image (**d**.). **f**., **g**. Conductance image (real part of the reflected microwave) of regions previously denoted by blue and red dots, respectively. The conductance image in **g**. shows a moiré pattern consistent with a twist angle of 0.7°.

SUPPLEMENTARY NOTE 3: INDEPENDENT CONFIRMATION BY SCANNING TUNNELING MICROSCOPY

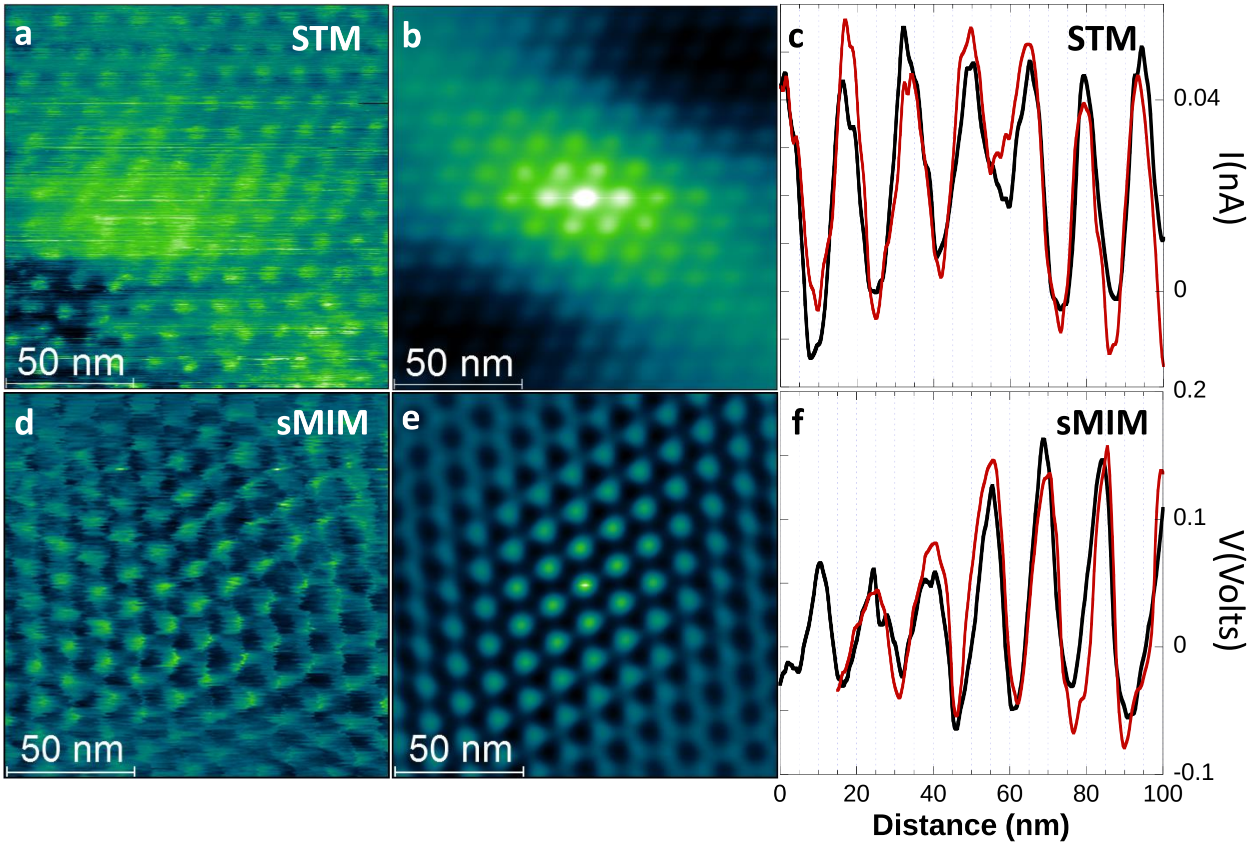

Supplementary Fig. 3. **Comparison between STM and sMIM data. a.** 150 nm x 150 nm STM current image (V_{sample} = -1.0 V, I = 0.5 nA), **b.**. its corresponding autocorrelation function and **c.** line profiles along the symmetry axis, **d.** 150 nm x 150 nm sMIM conductance image, **e.** its corresponding autocorrelation function, and **f.** line profiles along the symmetry axis.

SUPPLEMENTARY NOTE 4: FINITE ELEMENT METHOD CALCULATIONS

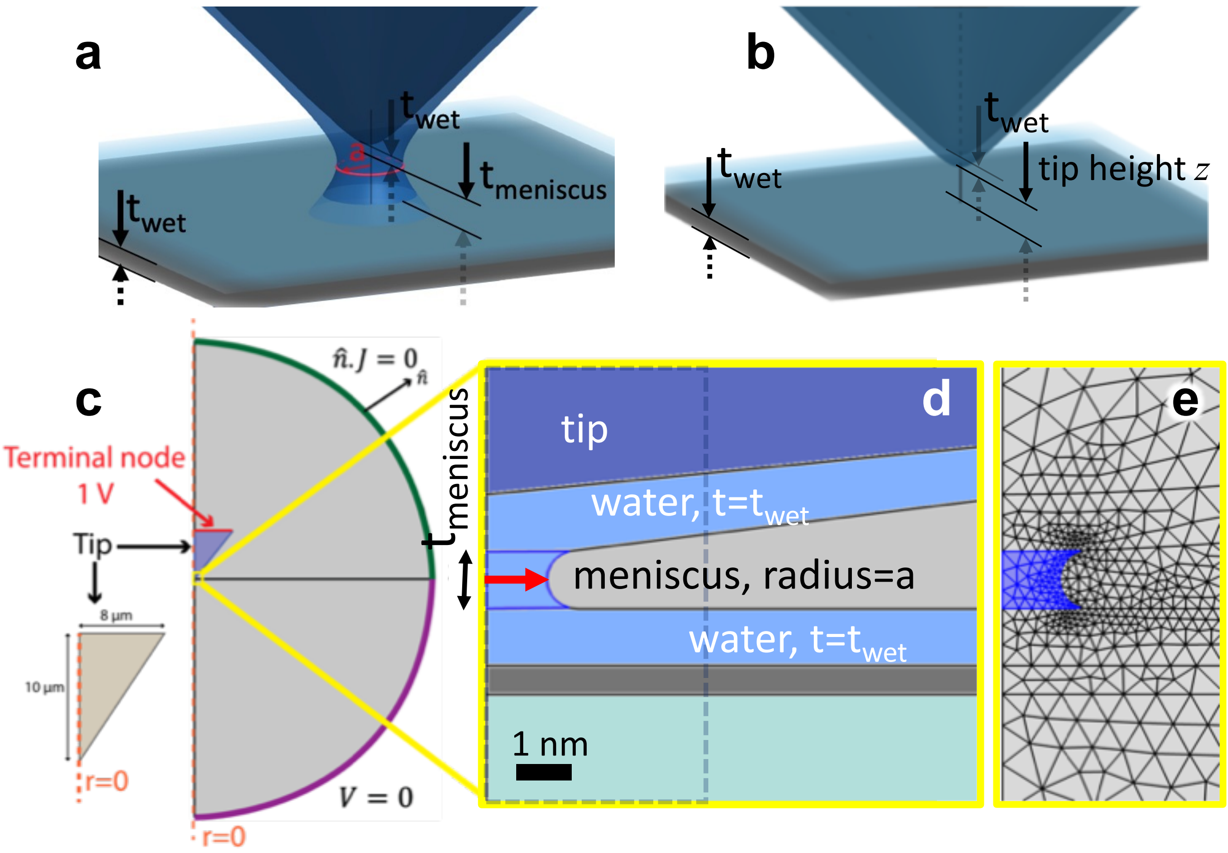

Supplementary Fig. 4. **Meniscus model. a.** Tip and surface connected by a water meniscus, as well as the native water layer present on surfaces. **b.** tip with no meniscus. The key factors for the model are the tip height z, the meniscus thickness $t_{meniscus}$, and the meniscus radius a. **c.** Boundary conditions imposed on the tip and sample for the evaluation of the real and imaginary parts of the reflected microwaves. **d.** detail of the model at the tip apex. The tip radius of curvature was assumed to be 50 nm. The native water layer present on surfaces is assumed to be t_{wet}=1 nm thick. The Twisted Bilayer Graphene layer is 0.7 nm thick, and a 10^6 S/m conductivity. **e.** detail of the meshing employed to evaluate the fields in the area defined by the dashed rectangle in **d.**

Aiming at fully understanding the effects observed in the measurements obtained by scanning Microwave Impedance Microscopy (sMIM), numerical simulations were performed implementing the Finite Element Method (FEM) [1] in the COMSOL™Multiphysics simulation tool, in order to calculate the tip-sample admittance.

The sMIM system was simulated in an axisymetrical two-dimensional environment, in

order to reduce the computational cost of the simulations [2]. The implemented tip has an inverted pyramid shape, with 8 μm at the top , 10 μm height, and 50 nm radius of curvature tip. The schematic diagram of the implemented simulation system can be seen in supplementary figure 4. In supplementary figure 4**a**, the meniscus thickness $t_{meniscus}$ was varied from 0.2 nm up to 8 nm, in accordance with reported values for menisci (see main text), for radii a of 1 (data not shown), 3 and 6 nm. As for the system without meniscus, supplementary figure 4**b**, the gap between the tip and the sample z was varied from 0.2 nm up to 1000 nm, resulting in the data shown in black dashed line in figure 2**b** of the main text.

Supplementary figures 4**c**, **d** and **e** show in more detail the simulation geometry and boundary conditions for the calculation of the impedance, and meshing diagram. The boundary conditions imposed in the system for proper evaluation of the impedance are shown in supplementary figure 4**c**, where the microwave current flows to the grounded substrate from the tip, which was a terminal node with a bias amplitude of 1V. The detailed structure of the tip and meniscus is shown in supplementary figure 4**d**, which assumes a metallic tip, a uniform layer of water on both tip and TBG surfaces of 1 nm thickness, a meniscus of thickness $t_{meniscus}$ and radius a, and twisted bilayer graphene sheet of conductivity σ=120 μS/m [3, 4], on top of a hBN flake 55 nm thick, with the relative permittivity ε_r=7, and σ=10^{-15} S/m. The simulations carried out in the two different scenarios, with (supplementary figure 4**a**) and without (supplementary figure 4**b**) water meniscus, used a mesh with 13,000 total elements, of which 1,500 corresponding to the elements in the boundary conditions.

At high frequencies the imaginary component of the admittance, the susceptance contribution, dominates since

$$C = \frac{Im(Y)}{\omega}, \tag{1}$$

where Y is the admittance, C is the capacitance, and $\omega = 2\pi f$ is the angular frequency at $f = 3$ GHz. But given the nanometer dimensions of the tip, and the impedance of the film, this complex association cannot be represented simply by a lumped element model described by equation 1. Thus, we calculated the real and the imaginary part of the admittance as a function of the permittivity and conductivity of the sample, assuming a 1 nm thick water meniscus of radius a = 1 nm, in addition to the water surface. The results are shown in supplementary figure 5, from which we can derive a few conclusions: a) the relationship

between the real component of the reflected microwaves and the sample conductivity is not single valued, except at its maximum around σ=11 S/m; b) both imaginary and real components are constant for σ<10^{-3} S/m and σ>10^3 S/m; and c) the dependence of both real and imaginary components on σ decreases with increasing ε. In essence, sMIM images contain significant amounts of data that are interrelated, and in order to evaluate the system's electronics properties we must solve the inverse problem, that gets considerably more complex as we include quantum effects, since they contribute to both conductivity and permittivity.

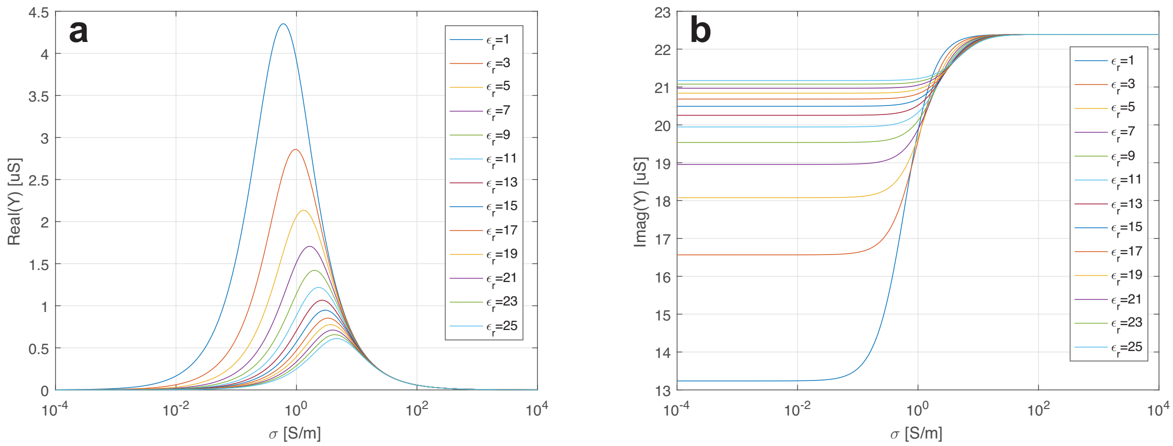

Supplementary Fig. 5. **a** Real and **b** Imaginary part of the admittance as a function of the conductivity and permittivity

SUPPLEMENTARY NOTE 5: RESOLUTION DEPENDENCE ON FILM CONDUCTIVITY

For the purposes of the current work, an important question is the dependence of the system spatial resolution on the sample conductivity. This is particularly important in spatially resolving the structures on twisted bilayer systems, such as the AA regions, AB/BA regions, and the domain wall SP region. We address this issue by performing calculations with cylindrical symmetry shown in supplementary figure 6. We examined three different conditions: a) a sample comprised of a central AA stacking region of 5 nm radius (see figure 1**a** in the main text for the experimental data) surrounded by an AB region (supplementary figures 6**a**,**e**; b) a sample comprised of region AB only (supplementary figures 6**b**,**f**); c) a sample comprised of hBN material only (supplementary figures 6**a**,**e**). The goal was to verify if the

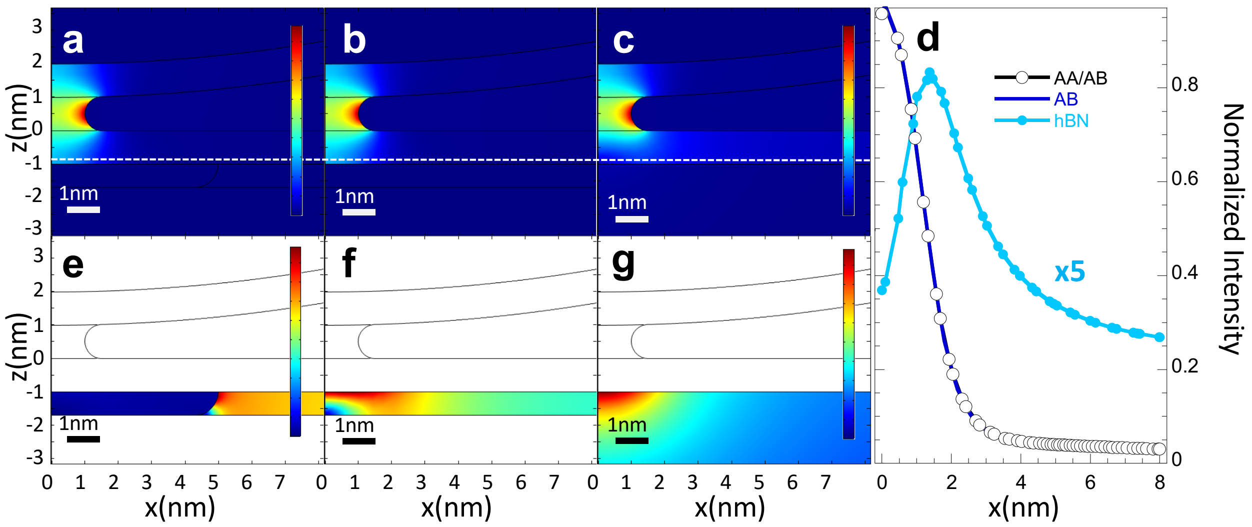

Supplementary Fig. 6. **Resolution dependence on surface conductivities, finite element calculations on the system tip-water meniscus-water layer-surface. The false color is keyed to the intensity of displacement field D. a.** Here, the surface was modeled as a circular region of radius 5 nm representing the AA sites, with a conductivity of $\sigma_{AA} = 3.6 \times 10^5$S/m, surrounded by AB region with a conductivity of $\sigma_{AB} = 1.2 \times 10^4$S/m. Full-scale (FS) of $\mathbf{D_{max}}$=0.2 C/m^2. **b.** Surface modeled by an uniform layer comprised exclusively of AB regions. FS $\mathbf{D_{max}}$=0.2C/m^2. **c.** Surface comprised of only hBN film, excluding TBG. FS $\mathbf{D_{max}}$=0.06C/m^2. **d. D** profile at the water-surface interface, at the water side, represented by the white dashed lines in **a.**, **b.** and **c.** maps; **e.** detail of **D** in the structure calculated in **a.**, focusing on the TBG layer. FS $\mathbf{D_{max}}$=2×10^{-6}C/m^2. **f.** detail of **D** in the structure calculated in **b.**, focusing on the TBG layer. FS $\mathbf{D_{max}}$=3.2×10^{-6}C/m^2. **g.** detail of **D** in the structure calculated in **c.**, focusing on the hBN layer. FS $\mathbf{D_{max}}$=2.3×10^{-3}C/m^2.

D field distribution changes as we change the local conductivity. We chose the following parameters for the conductivities [3, 4]: AA sites, with a conductivity of $\sigma_{AA} = 3.6 \times 10^5$S/m, AB sites with a conductivity of $\sigma_{AB} = 1.2 \times 10^4$S/m, and hBN with σ=10^{-15} S/m. Supplementary figure 6**d.** summarizes our findings, by displaying the displacement field **D** modulus profile, taken at the water layer just above the film of interest, similar to the results shown in figure 3 of the main text. We assumed a 1 nm thick water layer on both surfaces and a 1 nm radius, 1 nm thickness water meniscus. The spatial resolution can be inferred from the width of the displacement field **D** peak, and we find that for both AA:AB and AB cases, no

appreciable change in resolution is found. For the hBN layer, however, we end up finding the maximum of **D** displaced towards the periphery of the meniscus, thus forming a torus in cylindrical symmetry. Nevertheless, the resolution is roughly the same, considering a 20 orders of magnitude change in film conductivity. In summary, the key parameter responsible for concentrating the displacement field **D** is the water meniscus with its high relative permittivity.

SUPPLEMENTARY NOTE 6: NAP EXPERIMENTS

The nap experiments have a key goal to further substantiate the presence and use of the water meniscus as an imaging device. The term "nap pass" is used by Asylum Research that refers to an expression used in aviation "nap-of-the-earth flight," that describes flight made at low altitude which closely tracks the contours of the underlying terrain [9]. The experiment is performed in two steps: 1) one pass in contact or non-contact mode in order to extract the topography; and 2) a second pass at a predetermined height $\Delta\ z$ above the surface using the topography information acquired in the previous scan in order to maintain the tip-substrate distance constant. This protocol is executed in a line-by-line fashion, in order to avoid sample drift. We must emphasize that $\Delta\ z$ is actually the piezo displacement and will not correspond to the tip height with respect to the surface whenever a meniscus is present. For these cases, the meniscus will be simply subject to a normal force, and deformation will take place at both meniscus and cantilever.

The convenience of a flat sample is instrumental to allow a unequivocal assessment of the capacitance and conductance signal. For this particular sample, the majority of the contrast was measured in the conductance channel, suggesting a sample conductivity above 10 S/m, as seen in supplementary figure 5. The capacitance signal contains the meniscus information, and comparing its value in contact and lift mode permits us to assess a degree of meniscus deformation.

Supplementary figures 7**a** and **c** are expanded portions of figure 2**a** and **b** in the main text, to help visualize the capacitance decrease (black solid lines) and concurrent force (red solid lines) measurements during the lift mode. The filled circles in supplementary figure 7**c** represent the capacitance drops at different lift heights, evaluated from the sMIM capacitance images. The first observation is that although for small lifts the tip-retraction curve

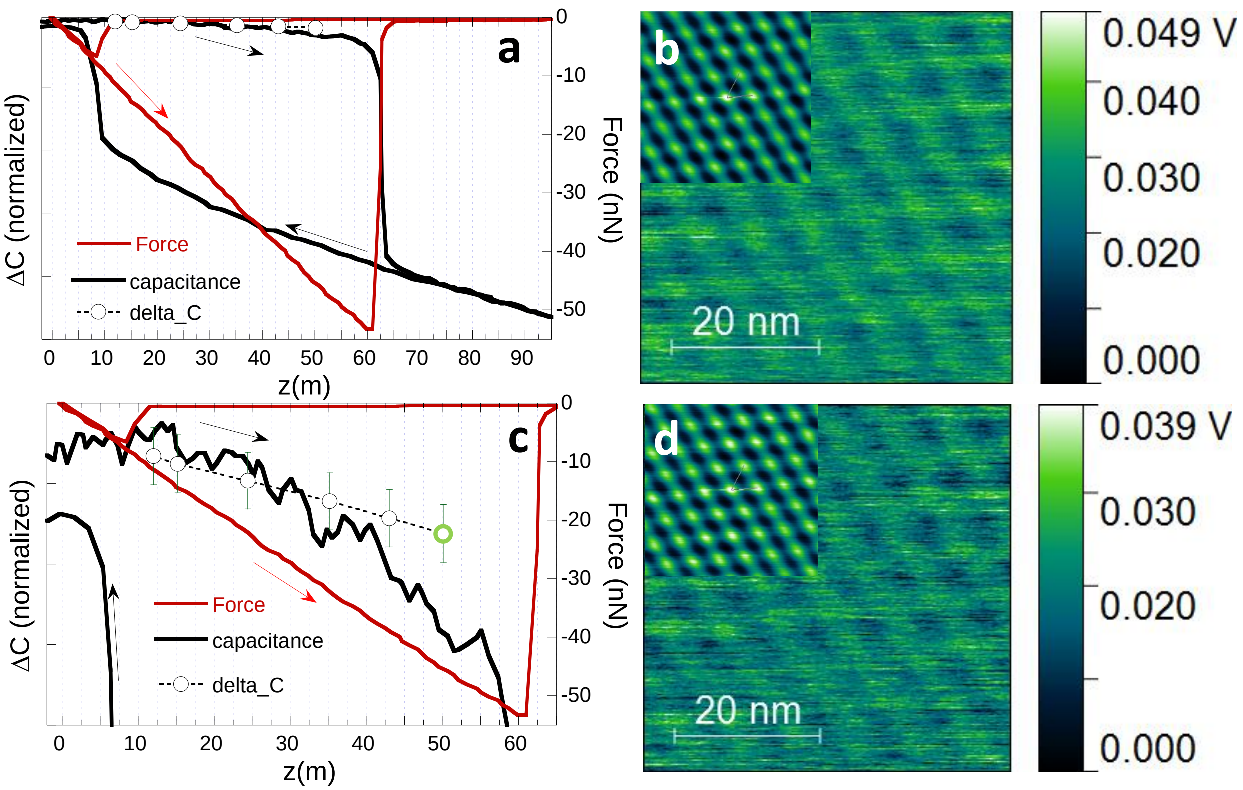

Supplementary Fig. 7. nap experiments: close up on figure 2 in the main text: a and **c** Force-curve (red line) and capacitance (black line) approach of a tip towards a TBG:hBN:Glass system. The empty circles correspond to capacitance changes evaluated from the difference in capacitance images taken in lift mode from the contact mode as a function of piezo displacement Δz; the error bars correspond to the Root-mean Square (RMS) values at each image; **b** conductance image in contact mode, **d** conductance image with the tip lifted during a piezo displacement Δz of 50 nm. The insets show the autocorrelation functions for both images.

(solid back line) is still in agreement with the capacitance change observed in lift mode, it does not decrease as notably. The interpretation for this difference is related primarily with meniscus formation dynamics [6–8]. During the lift scan, the tip stays at a constant height thus providing more time for meniscus nucleation. The expected impact on the capacitance difference is consequently a smaller decrease because of a higher volume meniscus, as observed. Examining the conductance channel images displayed in supplementary figures 7**b**, contact, and 7**d**, we observe that imaging is still adequately performed for a lift of 50 nm, ruling out the possibility of a metallic fragment or burr as the primary factor responsible for the resolution (also see the green circle in supplementary figure 7**c**). The inset shows

the auto-correlation functions, showing minimal changes in correlation lengths and same periodicity. The color scale nevertheless shows a decrease in signal amplitude for the image acquired in the lift mode, consistent with a more decoupled system because of a stretched meniscus.

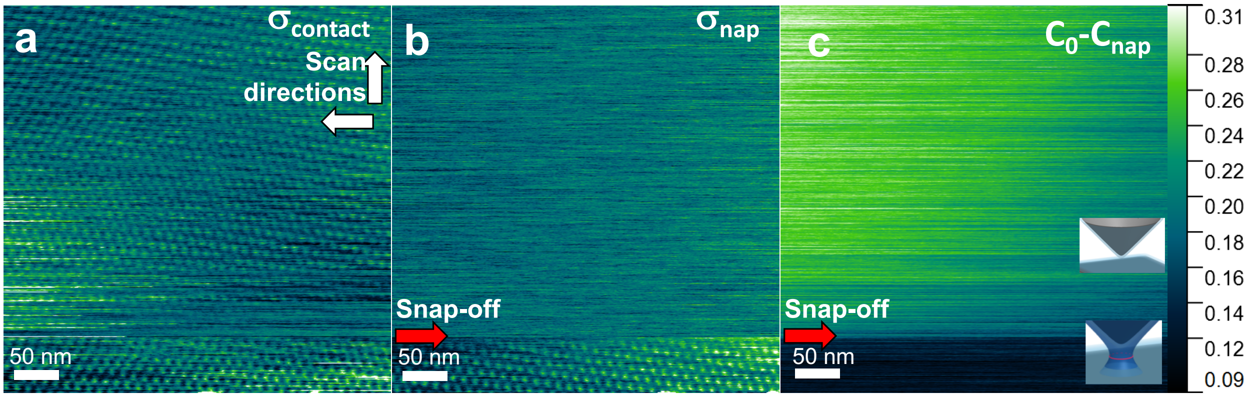

Supplementary Fig. 8. **Dynamics of meniscus nucleation and stability, and impact on imaging conditions. a.** Real part of the reflected microwave, or conductance, with tip in close proximity to surface but in the attractive regime. **b.** Real part of the reflected microwave with tip in the lift mode, with a piezo displacement Δz of 300 nm. At the red arrow the tip snaps off the surface due to meniscus rupture. **c.** Difference image of the imaginary component of the reflected microwave (capacitance) in close proximity (C_0) and in lift mode (C_{nap}). At the meniscus rupture, the capacitance difference increases, as C_{nap} corresponds to a larger spacing between tip-surface.

Another important aspect concerns the nucleation time of a meniscus and its stability. Szoszkiewicz [6] performed several studies aimed at the water meniscus nucleation processes. It was found that the meniscus nucleation process was thermally activated, and the time required to stabilize it would place an upper limit on the tip velocity above which it would not form. In essence, meniscus formation dynamics is dictated by relative humidity, temperature, normal force applied to the tip, and tip velocity. Above a certain critical velocity, which is a function of all of the above parameters, there is not enough time for meniscus nucleation, which is typically of the order of a few ms at room temperature and 37% humidity [6] At 299K and 39% humidity, the critical velocity reported was 1μm/s [6]. Choe [7] reported additional and intriguing results on the water meniscus, having observed meniscus as long as 12 nm (at 45% humidity), and with radius a as narrow as 1.3 nm (at 15% humidity and normal force of 600 pN).

We performed an additional study to identify the absolute limits of meniscus stability for our typical experimental conditions, focused primarily on the normal force applied to the tip, and tip velocity. Supplementary figure 8 shows our results, taken at T=293 K, relative humidity of 51%, tip velocity of 2μm/s, and a 300 nm lift (which would correspond to a 300 nN normal force). Supplementary figure 8**a** corresponds to the real part of the reflected microwave (or conductivity) image in close proximity, supplementary figure 8**b** conductivity image taken at 300 nm lift, and supplementary figure 8**c** is the capacitance difference image in close proximity (taken simultaneously with the data shown in supplementary figure 8 **a**) and 300 nm lift (taken simultaneously with the data shown in supplementary figure 8**b**). As indicated by the red arrow in supplementary figures 8**b**, and **c**, after a few line scans the meniscus snaps off, and imaging is only possible in close proximity (supplementary figure 8**a**). In essence, for the first few line scans the water meniscus was in a supercooled state, which eventually evaporates during lift. The capacitance difference signal ($C_0 - C_{nap}$) exhibits an increase after snap off, which is expected as the tip sits further away from the surface thus yielding a smaller C_{nap}.

References

[1] Volakis, J. L.,Chatterjee, A.,Kempel, L. C. *Finite Element Method Electromagnetics: Antennas, Microwave Circuits, and Scattering Applications.* Wiley Interscience, 1998.

[2] M. Koshiba and K. Inoue. Simple and efficient finite-element analysis of microwave and optical waveguides. *IEEE Transactions on Microwave Theory and Techniques*, 40(2):371–377, 1992.

[3] Xiao-Yong Fang, Xiao-Xia Yu, Hong-Mei Zheng, Hai-Bo Jin, Li Wang, and Mao-Sheng Cao. Temperature- and thickness-dependent electrical conductivity of few-layer graphene and graphene nanosheets. *Physics Letters A*, 379(37):2245–2251, October 2015. doi: 10.1016/j.physleta.2015.06.063. URL `https://doi.org/10.1016/j.physleta.2015.06.063`.

[4] M. Anđelković, L. Covaci, and F. M. Peeters. DC conductivity of twisted bilayer graphene: Angle-dependent transport properties and effects of disorder. *Physical Review Materials*, 2 (3), March 2018. doi:10.1103/physrevmaterials.2.034004. URL `https://doi.org/10.1103/`

physrevmaterials.2.034004.

[5] I. Glatt, A. Livnat, and O. Kafri. Direct determination of modulation transfer function by moiré deflectometry. *Journal of the Optical Society of America A*, 2(2):107, February 1985.

[6] Robert Szoszkiewicz and Elisa Riedo. Nucleation time of nanoscale water bridges. *Physical Review Letters*, 95(13), September 2005.

[7] H. Choe, M.-H. Hong, Y. Seo, K. Lee, G. Kim, Y. Cho, J. Ihm, and W. Jhe. Formation, manipulation, and elasticity measurement of a nanometric column of water molecules. *Physical Review Letters*, 95(187801), October 2005. doi:10.1103/physrevlett.95.187801. URL https://doi.org/10.1103/physrevlett.95.187801.

[8] Simon Carpentier, Mario S. Rodrigues, Miguel V. Vitorino, Luca Costa, Elisabeth Charlaix, and Joël Chevrier. Out of equilibrium anomalous elastic response of a water nano-meniscus. *Applied Physics Letters*, 107(20):204101, November 2015.

[9] Roger Proksch, Marta Kocun, Donna Hurley, Mario Viani, Aleks Labuda, Waiman Meinhold, and Jason Bemis. Practical loss tangent imaging with amplitude-modulated atomic force microscopy. *Journal of Applied Physics*, 119(13):134901, April 2016.
