# Supplementary material for: The limits of near field immersion microwave microscopy evaluated by imaging bilayer graphene moiré patterns
Source: Nat Commun. 2021 May 20;12:2980. doi: 10.1038/s41467-021-23253-2 (PMC8170674; doi:10.1038/s41467-021-23253-2)
Supplement: Supplementary file 3 — Description of Additional Supplementary Files [file 41467_2021_23253_MOESM3_ESM.pdf]

Description of Additional Supplementary Files

Supplementary Movie 1: Animation sequence of the displacement field D as the tip approaches the surface. This sequence culminates in the meniscus formation. Note that the color scale changes in order to help visualization.

Supplementary Movie 2: Animation sequence of the displacement field D as the tip approaches the surface. This sequence does not include meniscus formation. Note that the color scale changes in order to help visualization.
